# Effects of Doubling the Standard Space Allowance on Behavioural and Physiological Responses of Sheep Experiencing Regular and Irregular Floor Motion during Simulated Sea Transport

**DOI:** 10.3390/ani10030476

**Published:** 2020-03-12

**Authors:** Grisel Navarro, Ramazan Col, Clive J.C. Phillips

**Affiliations:** 1 Centre of Animal Welfare and Ethics (CAWE), School of Veterinary Science, University of Queensland, Gatton 4343, Australia; rcol@selcuk.edu.tr (R.C.);; 2 Departamento de Medicina Veterinaria, Facultad de Recursos Naturales, Universidad Católica de Temuco (UCT), Temuco 4780000, Chile; 3 Department of Physiology, Faculty of Veterinary Medicine, University of Selcuk, Campus 42035, Turkey

**Keywords:** animal behaviour, sheep, ship motion, space allowance, stocking density

## Abstract

**Simple Summary:**

Sheep are sometimes transported long distances by ship, and space allowance is one of the most contentious aspects of welfare provision. Previous research has not examined major increases in space allowances, so we investigated sheep responses to a doubling of the standard Australian allowance. Sheep were exposed to simulated ship motion of a regular or irregular nature, representing smooth sailing or high seas, or a control treatment without motion. Doubling space allowance provided evidence of reduced stress: less pushing each other, less need for the sheep to hold their heads close together, less leaning against their enclosure and lower heart rates and LF/HF ratio. It did, however, increase stepping by the sheep to correct their balance. Irregular motion reduced the time sheep spent chewing the cud, also evidence of stress, however, balance corrections were more common in regular motion, possibly because the sheep could predict the movement and step accordingly. We conclude that doubling sheep’s space allowance during simulated ship transport improves their welfare, and that irregular motion limits balance control and may reduce welfare.

**Abstract:**

Transporting livestock at high stocking density by ship presents significant risks to their welfare, especially if it is over long distances. Previous research has investigated small variations in density for long periods or a moderate variation for short periods. The objective of this study was to assess the effects of a doubling of space allowance during two types of simulated ship movement, regular and irregular floor motion, on the welfare of sheep for a short one-hour period. Six 25 kg sheep were restrained in pairs in a crate on a programmable platform that generated roll and pitch motion typical of that experienced on board ship. Sheep were subjected to regular or irregular movement or a control treatment at high and low stocking densities (0.26 and 0.52 m^2^/sheep) in a multilevel changeover design. Irregular movement was programmed as a sequence of 30 different amplitude and duration values for pitch and roll movements, which were randomly selected by computer software controlling the movement. Regular movement was the mean of these values, which represented approximately 33% of the recommended maximum tolerance for livestock carriers. Behaviour was recorded by six cameras positioned around the crate. The low space allowance increased sheep pushing each other (Low: 4.51 events/h, High: 1.37 events/h, *p* < 0.001), affiliative behaviour, with their heads one on top of the other (Low 8.64, High 3.75 s/h, *p* = 0.02) and standing supported by the crate (Low 96, High 3.2 s/h, *p* < 0.001). Sheep stepped more frequently when more space was provided, particularly in the forward (Low 6.4, High 8.4 steps/h, *p* = 0.02) and left (Low 4.0, High 4.7 steps/h, *p* = 0.03) directions. The low space allowance group also had i heart rates, providing evidence of physiological stress. Irregular movement reduced rumination (Irregular 288, Control 592, Regular 403 s/h, *p* = 0.02), which was evidence of reduced welfare, but balance corrections by stepping were more common if the motion was regular. Thus, there was evidence that the low space allowance increased interactions between sheep and was stressful, and that irregular floor motion in simulated ship transport limited balance control and reduced welfare.

## 1. Introduction

An increasing number of livestock are transported every year around the world, for both breeding and production of meat [1,2,3]. Over the last thirty years transport by sea has become an increasingly common way to move sheep over long distances [1,2]. However, there is scientific and public concern about the welfare of the animals on board [1,4]. Space allowances are the most controversial aspect concerning the animal’s welfare, because providing sufficient space for normal behaviour has a high economic cost [5,6,7,8]. Under the current commercial conditions, space allowances are little more than the physical space occupied by the animals, which reduces the unit cost of transport [7]. Transported sheep may not even receive recommended space allowances; in one study [9] over 30% of 6578 sheep transported by road were at higher densities than the UK’s Farm Animal Welfare Council recommendations [10]. Insufficient space limits their behavioural freedom, in particular opportunities to lie down, in both trucks [11] and ships [12]. Stock become frightened of falling and not being able to get up again if other animals have occupied their space. Balance control is therefore crucial at high stocking densities.

Space allowance interacts with many aspects of the animal’s environment [6], especially their ability to keep their balance. In one study, sheep transported by road at a low space allowance (0.39–0.78 m^2^/animal) had more losses of balance and slips than those at higher space allowances (1.04–1.56 m^2^) [13]. Sheep prefer to stand independently during the transport and not to touch each other. During sea transport, sheep have to address more varied movements (heave, sway, surge, yaw, pitch and roll) than during road transport, and the animal´s responses to these movements are poorly understood [2]. Pitch, roll and heave are the most common motions, associated with loss of balance and probably motion sickness [14]. Heave and roll increase sheeps’ heart rates, demonstrating greater arousal, than pitch [15]. Their main strategy to maintain balance on a moving floor involves stepping with their forelimbs frontwards and backwards, especially when they are exposed to heave and roll motions [16].

Sheep should be given sufficient space for postural adjustment during road transport [8,13], but little is known about the appropriate space and its association with the balance maintenance strategies during transport by sea. We previously compared the impact on sheep behaviour and heart rate of increasing Australian standard allowances for sheep in ships (0.26 m^2^/sheep) by providing an extra 15% or 35% of space. Both levels of extra space increased lying, but only the biggest space allowance reduced aggression and increased heart rate variability, suggesting reduced stress [17]. The biggest allowance also reduced pushing behaviour and stepping to correct balance, as long as the motion was regular. Given that the responses to extra space are dependent on how much extra space is given and the regularity of the movement, this research investigated sheep responses to an extra 50% of space, compared with the Australian standards, in simulated sea transport with regular and irregular motion.

## 2. Material and Methods

The study was conducted at the Gatton campus of the University of Queensland (27.3° S, 152.2° E), under the Animal Ethics Committee’s approval number SVS/CAWE/156/15/UQ SVS.

### 2.1. Animal Housing and Management

Six Merino–Dorper crossbred sheep of age 4 months and weight (mean ± SEM) 25.0 ± 0.7 kg were used for the experiment. During the study the animals remained alone in a paddock of approximately 20 × 10 m, except for the time when they were exposed to the treatments in an experimental facility at the Queensland Animal Science Precinct. They were fed twice daily, in the morning and afternoon, with ad libitum lucerne hay and chaff and 2 kg/head/d of lucerne pellets. They had ad libitum access to water in the paddock. Three weeks before the first trial started they were exposed to a habituation process, in which they were trained using only positive reinforcement twice daily over a 32 d period to accept human presence, then handling, and finally to enter the crate in which they would be restrained for the movement studies. The main habituation processes were as follows, which occurred until the sheep failed to respond visibly to the stimulus but could occur simultaneously or consecutively, as deemed best for the end goal of habituation to all stressors. Sheep were first offered pellets by hand, every 2 h for 10 d, then loaded and unloaded over 8 d, heart rate monitor attaching over 7 d and finally spending 3–4 h in the research facility over 20 d. The sheep were exposed in pairs (combinations detailed below) to two space allowances and three different movement treatments: irregular movement, regular movement and a control treatment with no movement, in a two factor factorial changeover design.

### 2.2. Simulating Sea Transport Motions

**Figure 1:**
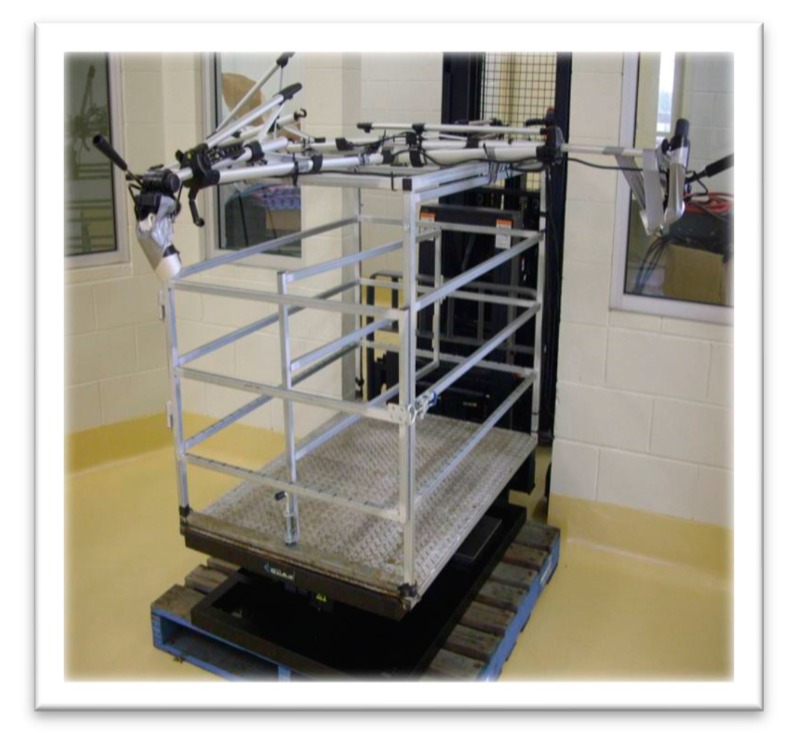
Platform and crate used to expose sheep to simulated ship movement.

An electronic platform (Model T2sMP, CKAS Mechatronics Pty Ltd., Melbourne, Australia) capable of reproducing roll and pitch motion [14] of similar magnitude to that normally experienced at sea was used in this study (Figure 1). The platform movements were programmed with Microsoft Visual Studio Solution C++ Express 2008 software, and a crate designed to hold sheep secured on top of the platform. The crate (0.87 m wide × 1.2 m long × 0.95 m high) was composed of four sides each constructed with 3 tubular steel bars. For the low space allowance, the crate was modified internally so that the shape of the crate was maintained (Length = Width × 1.468) but the space allowance was reduced to 0.52 m^2^, which was 50% of the larger space, 1.04 m^2^. The new dimension was 0.60 m wide, 0.88 m long and 0.95 m high. Steel bars, identical in dimension, position and number to those of the existing crate, were used to diminish its size. The floor of the crate was of steel, diamond plate sheeting, providing an antislip footing for the sheep.

### 2.3. Experimental Design

Six sheep were exposed for one hour periods in pairs to six treatments in a replicated 6 × 6 Latin square design, incorporating the two factors Space allowance and Motion. Each sheep therefore was exposed to each of the six treatment combinations four times. Pairs were selected from the six sheep using another Latin square design to ensure equal use of each animal, with no animal exposed to treatments two times in one day. The first factor was motion type applied to the platform: a motionless control treatment (C), irregular (I) motion, programmed as 30 randomly-selected amplitude and duration values of combined pitch and roll motions from those possible, and regular (R) motion, the mean value of these pitch and roll motion values applied to the movement of the platform. These mean motion values represented approximately 33% of the recommended maximum tolerance for livestock carriers [14]. The second factor was space allowance: low space allowance (L: 0.26 m^2^/sheep, which is the minimum space allowance for a sheep of 28 kg specified by Australian standards, ASEL, 2011) [18] and high space allowance (H: 0.52 m^2^/sheep, a doubling of space compared with L).

### 2.4. Behavioural Parameters

#### Behaviour Recording

Six video cameras (model K-32HCVF, Ashmore, QLD, Australia) were located around the crate and sheep behaviour was recorded in real time for every treatment application. A digital video recorder (Kobi H.266, Model XQ-L 900H, Ashmore, QLD, Australia) was used to record images, and behaviours were coded from a continuous recording of each animal using purpose-made software (Cowlog 3.0.2, University of Helsinki, Helsinki, Finland). The software separately identifies the number of events of each behaviour and the total duration [19]. During replay, videos were coded by a single researcher (RC), who was trained in video analysis at the Centre for Animal Welfare and Ethics. Four 5 min scan samples were observed/h (at 0–5, 18–23, 36–41 and 55–60 min, termed Periods 1–4). The mutually-exclusive head positions recorded as durations were down, middle, or up, relative to the horizontal position of the head when it was not against the bars of the cage, up or down when it was against the bars of the cage, above another sheep, or turned around. Durations of lying, ruminating and standing (with and without being supported by leaning against the crate) were recorded. The frequency of pushing (a deliberate attempt to displace a conspecific with the head, shoulders or side of the body) and aggression (a rapid and threatening attack to a conspecific with the head, or a kick or butt) events was recorded. Videos were separately reviewed by a separate researcher (GN, also trained at the Centre for Animal Welfare and Ethics) to classify the number of steps of the fore and hind limbs in the following directions in each 5 min period: forward, backward, to the right, to the left, diagonal forward left, diagonal forward right, diagonal back left, diagonal back right, and returning to the same place.

### 2.5. Physiological Parameters

#### Heart Rate and Its Variability

Heart rate monitors (Polar S810i, Kempele, Finland) were attached to the thorax of one of each pair of sheep, which was randomly selected during each treatment application. The device included an electrode belt, with two electrodes fitted around the thorax of the sheep. Four segments of 512 beats (approximating to the four 5 min periods described above) were taken from each exposure to treatment, using time and frequency domain analysis. The data were analysed using Kubios HRV 2.1 software [20], selecting the Low (LF) and High (HF) frequency bands widths according to the recommendations for sheep (LF: 0.04–0.2 Hz, HF: 0.2–0.4 Hz) [21].

### 2.6. Statistical Analysis

General linear multilevel regression models were used to analyse the effects of the treatments. Each behaviour was analysed for significance of animal as a random factor and the following fixed factors: period, space allowance (Low and High), motion type (R, I and C), and the interaction between the two treatment factors. A normal distribution of residuals from the model was verified with the Anderson–Darling test, if necessary following data transformation by log_10 +1_ (head turned around; standing against crate, pushing; aggression; all stepping data), square root (head against bars, down and up); or Box Cox [22] (all heart rate data). Back-transformed values are provided. A second general linear model analysed the 16 different stepping directions with the following fixed factors: period, space allowance (L and H), motion (R, I or C) and the following interactions: space allowance* motion; period* space allowance; period*motions and space allowance*motions* period, with animal again included as a random factor. Tukey´s and Fisher’s post hoc comparison tests were used to identify which means were significantly different from each other. For heart rate (HR) data, a general linear model of the parameters extracted from 512 beat segments by the Kubios software investigated the following HR variability measures as well as HR mean: RR-mean (R wave to R wave interval, i.e., the inverse of HR); RR-SDNN (standard deviation of the RR intervals); RMSSd: root mean square of the successive differences and LF/HF: ratio of low to high frequency beats. The model had the following fixed factors: day, companion and treatment, with animal as a random factor. We compared two commonly used methods of analysing spectral frequency components as integrals of power spectrum density over specific bands (Fast Fourier transformation, FFT) and components determined by autoregressive algorithms (Autoregressive method, AR). We present FFT values as these were most informative [23]. The statistical package Minitab 17 (Minitab Inc., State College, PA, USA) was used for the analyses.

## 3. Results

### 3.1. Behaviour

There were no significant interactions between space allowance and motion type, so results are detailed separately. Sheep spent more time with their head above their partner in Low (8.64 s/h) compared with High (3.75 s/h) space allowance (*p* = 0.02) (Table 1). There was no significant effect of the head position being either on the bars or not, but there was a tendency for sheep in the Low space allowance to spend longer with their heads turned around (*p* = 0.07). Sheep spent more time supported against the crate in Low than High (96 and 3.2 s/h respectively) space allowance (*p* < 0.001). There was no difference in the time that the animals spent lying down or ruminating in the Low or High space allowances. The number of pushing events was greatly increased in Low space allowance (*p* < 0.001), but there was no difference in the number of aggressive events between the Low and High space allowances. Stepping responses were affected independently by space allowance, movement and period. Sheep stepped more in the forward and left directions and less to the same place when more space was provided (Table 2).

Motion type did not have any significant effects on the behaviour of the sheep, except that time spent ruminating was reduced in the Irregular motion, compared with the Regular and Control treatments, there tended to be more pushing in the motion treatments compared with the Control (Table 3) (*p* = 0.07), and there were major effects on stepping (Table 4). During the regular movements the sheep stepped more in all directions compared with irregular and when there was no movement (Appendix A Table A1). In addition, all the stepping directions movements, except diagonal back right, were increased in the initial period of each treatment (0–5 min) (Appendix A Table A2).

### 3.2. Physiological Measures

The Low space allowance treatment increased the HR mean, the LF beats, and it decreased the HF beats, compared with the High treatment (Table 4). As a result, the ratio of LF/HF beats was increased in the Low space allowance. There were interactions between the space allowance and motion treatments for the RR_SDNN (ms) and RMSSD values (*p* = 0.01 and 0.03 respectively) (Table 5). RR_SDNN was reduced in the High space allowance, compared to the Low space allowance, but only in the Control treatment (83.4 vs. 43.9), not in the Regular treatment (49.8, 40.2) or the Irregular treatment (54.2, 80.2). The RMSSD was increased by the High space allowance, compared with the Low, in the Regular treatment (15.6 vs. 11.5), but not in the Control (12.4, 14.2) or Irregular (16.2, 14.0) treatments.

## 4. Discussion

This study supports our previous finding [17] that a low space allowance (0.26 m^2^/head = ASEL standards) and exposing sheep to a sequence of motions (both Regular and Irregular) have an impact on the welfare of sheep in simulated ship transport. The sheep responses included increased agonistic behaviour (pushing), stepping behaviours, heart rate and LF/HF ratio in the Low space allowance, evidence of increased stress.

Compared with sheep in the high space allowance, sheep in the low space allowance showed more affiliative behaviour and doubled the time spent with their head above their partner (8.64 s/h v/s 3.75 s/h, *p* = 0.02). This social attachment could be related to sheep attempting to stay close together under stress, as a gregarious species, protecting themselves as a pair when they have to face a stressful condition. It must be acknowledged that such affiliative behaviour is facilitated by closer proximity at the low space allowance, and further research could investigate the motivation for such behaviour. It has been observed previously [15,24] and suggests that having their head above their partner’s reduced the negative emotion or discomfort caused by lack of space.

Sheep also increased pushing in the Low space allowance (4.51 vs. 1.37 pushes/h in the High space allowance). This increase in pushing behaviour can be seen as a consequence of competition for space, particularly for postural adjustment during their attempts to maintain their balance. In pigs an increase in social interaction has been associated with stress [25], with more social interactions at low space (2.0 m^2^/head) compared with higher space allowances (2.4–4.8 m^2^/head). Other research has shown an increase in negative social interactions, including pushing, in dairy ewes when less space was provided (1 m^2^/ewe, compared with 2 and 3 m^2^/ewe) [26]. The increase in social interactions might also result from the closer physical proximity of neighbouring sheep in reduced space allowance, without any involvement of stress. However, the heart rate responses to low space (0.26 m^2^/head), in particular the increase in HR rate and LF/HF ratio and the reduction in HF, support the hypothesis that the sheep were stressed as a result of lack of space.

Simulated sea motion appeared to exacerbate the responses, the pushing responses tended to be increased when sheep were exposed to motion (irregular and regular) compared with the control with no motion (I = 9.82, R = 8.04 and C = 5.30 n/h; *p* = 0.07). Aggression was not affected by space allowance (*p* = 0.20), in contrast to our previous research [17] in which aggression increased at low (5.9 times/h) and medium (5.6 times/h) space allowances, compared with high space allowance (3.9 times/h). The results of the current study may be different due to the smaller group size of two instead of three. If the group size increases (using the same m^2^/head), the amount of space to be shared among the animals (group space) also decreases, making it insufficient to allow the performance of certain interactive behaviours, such as aggression [5]. It is also possible that the sheep had habituated to each other after the first trial of this sequence of two consecutive similar trials, having created a confirmed dominance order, making aggressive interaction unnecessary. Reuse of animals between experiments helps to reduce the number of animals exposed to stressful procedures.

Sheep spent more time standing against the crate support when they were exposed to low space allowance. Sheep prefer to travel independently and keep their balance by supporting their body against a vehicle [13]. These results are consistent with our previous trial [17], in which sheep increased their time standing against the crate when less space was provided (0.26 m^2^/sheep), compared with medium (0.30 m^2^/sheep) and high (0.35 m^2^/sheep) space allowances. Increased stepping in the high space allowance reflects more opportunity to step to keep their balance without interfering with the personal space of the other animal. The increase in stepping in the regular movement suggests that prediction of the floor position occurred and that this facilitated stepping behaviour.

The decrease in rumination time produced by irregular motion compared with regular and no motion has been observed previously [17]. A reduction in rumination behaviour has also been previously described in deer during transport by road compared with those remaining stationary [27], and it is likely that reduced rumination reflects an increase in stress.

This study has limitations because it only addresses space allowance effects under simulated not actual travel, only in small groups of sheep compared to those transported commercially, and only for short periods of time compared to commercial transport. In addition, sheep were accustomed to the apparatus beforehand, whereas commercially-transported sheep are not accustomed to the ship and this could contribute to stress experienced on board. This was deliberate in our study to avoid any complications of neophobia on the effects of space allowance and motion. However, the very different conditions on board commercial transport vessels must be recognized. In commercial transport, the close proximity of an unknown conspecific may not motivate a sheep to put its head under or over its head, whereas our sheep’s familiarity with each other may facilitate that behaviour. Secondly, the vertical distance moved by sheep will depend on where they are on a ship (those in the metacentre move the least, those at the edges of the deck much more). When we rotated the platform, we used a proportion of 33% of the standard tolerances for actual ships, in terms of the angle through which they are required to tolerate when rotated during heavy seas. The study therefore attempts to identify a proof of concept for fundamental changes in behaviour, stress levels and heart rate variables. These should be investigated in commercial transport situations before any conclusions about the welfare of sheep on board are drawn.

Other limitations include that the observer was not blind to treatments, impossible in this study because of the different sizes of the pens. Furthermore, using just a single observer meant that we could not measure interobserver reliability. The best procedure is to have several observers all proven statistically to be coding behaviour in the same way, but labour availability precluded that in this study. We also chose not to have the same observer view video footage twice, which would have given us a measure of interobserver reliability; this was because of the large amount of footage that already needed to be observed. We also acknowledge that the models we constructed assume independence in behaviours. For many of behaviours that we measured this was not likely to be the case, e.g., stepping in different directions, lying and standing.

## 5. Conclusions

A low space allowance, equating to the minimum specified by Australian standards, produced stress and reduced the welfare of sheep during simulated sea conditions, compared with a much greater space allowance, that was twice the area. Providing the extra space also seemed to provide more opportunity for sheep to step to keep their balance, particularly if the floor motion was regular and could be predicted. Extra space therefore benefits sheep welfare.

## Figures and Tables

**Table 1:** Effects of Low and High space allowance treatments on the behaviour of sheep during simulated sea conditions.

Behaviour	Low	High	SED	F-Value	*p*-Value
HEAD POSITION					
Head not against bars					
Down (s/20 min)	164	189	74.18	0.27	0.61
Middle (s/20 min)	473	563	157.9	0.82	0.37
Up (s/20 min)	115	195	89.84	1.99	0.17
Head against bars					
Down (√s/20 min)	9.50	8.41	3.776	0.36	0.55
(s/h)	271	212			
Up (√s/20 min)	10.7	11.2	5.251	0.04	0.84
(s/h)	342	375			
Above partner (log_10_ + 1 s/20 min)	0.46	0.09	0.312	5.87	0.02
(s/h)	8.64	3.75			
Turned around (log_10_ + 1 s/20 min)	1.30	1.06	0.282	3.39	0.07
(s/h)	60	35			
STANDING					
Against crate (log10 + 1 s/20 min)	1.51 ^a^	0.03 ^b^	0.465	25.53	<0.001
(s/h)	96.9	3.21			
No support (s/20 min)	419	584	156.4	2.82	0.10
Total standing (s/20 min)	537	603	256.2	0.29	0.59
OTHER BEHAVIOUR					
Lying (s/20 min)	620	649	184.3	0.06	0.80
Ruminating (s/20 min)	409	446	146.3	0.16	0.68
Pushing (log_10_ +1 n/20 min)	0.654 ^a^	0.138 ^b^	0.470	23.97	<0.001
(n/h)	4.51	1.37			
Aggression (log_10_ + 1 n/20 min)	0.13	0.05	0.133	1.71	0.20
(n/h)	4.08	3.39			

^a,b^ Means with different superscripts differ significantly by Tukey’s comparison test.

**Table 2:** Significant (*p* < 0.05) effects on sheep (*n* = 6) stepping in different directions in the low and high space allowance treatments in simulated sea conditions.

Stepping Directions	Space Allowance		SED	F-Value	*p*-Value
	Low	High			
Forward (log_10_ + 1 n/20 min)	0.326	0.445	0.237	5.79	0.02
(n/h)	6.36	8.37			
Left (log_10_ + 1 n/20 min)	0.128	0.197	0.152	4.72	0.03
(n/h)	4.05	4.74			
Same place (log_10_ + 1 n/20 min)	0.131	0.060	0.106	10.1	0.002
(n/h)	4.05	3.45			

**Table 3:** Effects of motion treatment (Control, Irregular and Regular) on the behaviour responses of sheep during simulated sea conditions.

Behaviour	Control	Irregular	Regular	SED	F-Value	*p*-Value
HEAD POSITION						
Head not against bars						
Down (s/20 min)	124	224	182	0.27	1.75	0.19
Middle (s/20 min)	495	530	529	0.82	0.07	0.94
Up (s/20 min)	200	103	163	1.99	1.07	0.35
Head against bars						
Down (√s/20 min)	6.86	9.31	10.7	0.36	1.95	0.15
(s/h)	141	260	343			
Up (√s/20 min)	10.6	9.74	12.4	0.04	0.45	0.64
(s/h)	339	285	463			
Above partner (log_10_ + 1 s/20 min)	0.23	0.22	0.39	5.87	0.63	0.54
(s/h)	5.04	5.02	7.36			
Turned around (log_10_ + 1 s/20 min)	1.07	1.12	1.37	3.39	2.33	0.11
(s/h)	34.8	39.5	70.81			
STANDING						
Against crate (log_10_ + 1 s/20 min)	0.427	0.735	0.615	5.87	0.84	0.44
(s/h)	8.02	16.30	12.4			
No support (s/20 min)	524	442	537	156.4	0.38	0.69
Total standing (s/20 min)	452	529	729	256.2	2.17	0.12
OTHER BEHAVIOUR						
Lying (s/20 min)	623	648	633	184.3	0.02	0.98
Ruminating (s/20 min)	592 ^a^	288 ^b^	403 ^a^	146.3	4.22	0.02
Pushing (log_10_ + 1 s/20 min)	0.25	0.43	0.52	0.470	2.81	0.07
(n/h)	5.30	8.04	9.82			
Aggression (log_10_ + 1 n/20 min)	0.02	0.09	0.16	0.133	2.21	0.12
(n/h)	3.15	3.70	4.43			

^**a,b**^ Means with different superscripts differ significantly by Tukey’s comparison test.

**Table 4:** Significant effects (*p* < 0.05) of low and high space allowance treatments on heart rate and its variability on sheep (*n* = 6) in simulated sea conditions.

Heart Rate Variable	Space Allowance		SED	F-Value	*p*-Value
	Low	High			
Heart rate (Box-Cox beats/min)	11.2	11.8	0.767	5.78	0.02
(beats/min)	141	125			
FFT_LF (Box-Cox), no units	33.83	27.41	7.219	5.84	0.02
Back-transformed values	82.9	77.3			
FFT_HF (Box-Cox), no units	7.9	12.4	0.191	8.43	0.004
Back-transformed values	1.68	1.89			
FFT_LF/HF * (Box-Cox)	7.43	6.48	1.316	10.2	0.002
Back-transformed values	12,500	6607			

* FFT_LF/HF = Fast Fourier Transformed ratio of low to high frequency interbeat intervals.

**Table 5:** Significant (*p* < 0.05) interactions between control, regular and irregular motion treatments and space allowance on sheep (*n* = 6) heart rate RR_SDNN and RMSSD in simulated ship transport.

Motion	Control		Regular		Irregular				
Space Allowance	Low	High	Low	High	Low	High	SED	F-Value	*p*-Value
^†^ RR_SDNN (m/s)	83.4 ^a^	43.9 ^b^	40.2 ^b^	49.8 ^a,b^	80.2 ^a^	54.2 ^a,b^	0.024	4.79	0.01
^‡^ RMSSD	14.4 ^a,b^	12.4 ^b,c^	11.5 ^c^	15.6 ^a,b^	14.0 ^a,b,c^	16.2 ^a^	0.159	3.63	0.03

^†^ RR_SDNN ^=^ Standard deviation of all interbeat intervals (IBIs) in the data set. ^‡^ RMSSD = The square root of the mean of the sum of the squares of differences between successive IBIs; ^**a,b,c**^ Means with different superscripts differ significantly by Tukey’s comparison test.

## Appendix A

**Table A1:** Effects of motion type on total stepping directions of sheep (*n* = 6) during simulated sea conditions.

Stepping Directions	Movements			SED	F-Value	*p*-Value
	Control	Regular	Irregular			
Forward (log_10_ + 1 n/20 min)	0.27 ^b^	0.53 ^a^	0.35 ^b^	0.237	9.24	<0.001
(n/h)	5.64	10.3	6.72			
Back (log_10_ + 1 n/20 min)	0.26 ^b^	0.52 ^a^	0.36 ^b^	0.239	8.44	<0.001
(n/h)	5.52	9.93	6.99			
Right (log_10_ + 1 n/20 min)	0.06 ^b^	0.22 ^a^	0.10 ^b^	0.115	15.6	<0.001
(n/h)	3.42	4.98	3.75			
Left (log_10_ + 1 n/20 min)	0.07 ^b^	0.29 ^a^	0.12 ^b^	0.152	15.7	<0.001
(n/h)	3.54	5.82	4.05			
Same place (log_10_ + 1 n/20 min)	0.08 ^a,b^	0.14 ^a^	0.07 ^b^	0.106	3.54	0.03
(n/h)	3.60	4.11	3.54			
Diagonal forward left (log_10_ + 1 n/20 min)	0.08 ^b^	0.20 ^a^	0.10 ^b^	0.130	7.85	<0.001
(n/h)	3.60	4.83	3.81			
Diagonal forward right(log_10_ + 1 n/20 min)	0.08 ^b^	0.19 ^a^	0.09 ^b^	0.135	6.04	0.003
(n/h)	3.63	4.68	3.72			
Diagonal back-left (log_10_ + 1 n/20 min)	0.02 ^b^	0.10 ^a^	0.06 ^a,b^	0.089	5.67	0.004
(n/h)	3.15	3.81	3.48			
Diagonal back-right (log_10_ + 1 n/20 min)	0.006 ^b^	0.09 ^a^	0.04 ^b^	0.073	10.3	<0.001
(n/h)	3.03	3.72	3.36			

^a,b^ Means with different superscripts differ significantly by Tukey’s comparison test.

**Table A2:** Significant interactions between total stepping directions and time period (minutes 0–5, 18–23, 36–41 and 55–60) on sheep (*n* = 6), during 1 h in simulated ship transport.

Stepping Directions	Period (min)				SED	F-Value	*p*-Value
	0–5	18–23	36–41	55–60			
Forward (log_10_ +1 n/20 min)	0.66 ^a^	0.31 ^b^	0.24 ^b^	0.31 ^b^	0.237	15.1	<0.001
(n/h)	13.7	6.12	5.19	6.12			
Back (log_10_ + 1 n/20 min)	0.67 ^a^	0.27 ^b^	0.23 ^b^	0.34 ^b^	0.239	16.0	<0.001
(n/h)	14.0	5.58	5.07	6.54			
Right (log_10_ + 1 n/20 min)	0.24 ^a^	0.10 ^b^	0.08 ^b^	0.07 ^b^	0.115	10.7	<0.001
(n/h)	5.19	3.75	3.60	3.51			
Left (log_10_ + 1 n/20 min)	0.28 ^a^	0.14 ^b^	0.11 ^b^	0.10 ^b^	0.152	7.23	<0.001
(n/h)	5.70	4.14	3.84	3.75			
Same place (log_10_ + 1 n/20 min)	0.14 ^a^	0.08 ^a,b^	0.05 ^b^	0.09 ^a,b^	0.106	2.87	0.04
(n/h)	4.14	3.60	3.36	3.69			
Diagonal forward left (log10 + 1 n/20 min)	0.23 ^a^	0.10 ^b^	0.08 ^b^	0.09 ^b^	0.130	6.39	<0.001
(n/h)	5.07	3.75	3.60	3.69			
Diagonal forward right(log10 + 1 n/20 min)	0.24 ^a^	0.06 ^b^	0.09 ^b^	0.09 ^b^	0.135	7.97	<0.001
(n/h)	5.19	3.42	3.69	3.69			
Diagonal back-left (log10 + 1 n/20 min)	0.13 ^a^	0.07 ^a,b^	0.01 ^b^	0.03 ^b^	0.089	8.00	<0.001
(n/h)	4.02	3.51	3.06	3.21			

^a,b^ Means with different superscripts differ significantly by Tukey’s comparison test.
